# Irisin and markers of metabolic derangement in non-diabetic Caucasian subjects with stage I-II obesity during early aging

**DOI:** 10.1371/journal.pone.0229152

**Published:** 2020-02-18

**Authors:** Jonica Campolo, Ettore Corradi, Alice Rizzardi, Marina Parolini, Cinzia Dellanoce, Maria Luisa Di Guglielmo, Patrizia Tarlarini, Marina Cattaneo, Maria Giovanna Trivella, Renata De Maria

**Affiliations:** 1 CardioThoracic and Vascular Department, CNR Institute of Clinical Physiology, Milan, Italy; 2 Clinical Nutritional Unit, Medical Department, ASST Grande Ospedale Metropolitano Niguarda, Milan, Italy; Universita degli Studi di Bari Aldo Moro, ITALY

## Abstract

Irisin concentrations are decreased in subjects with overt diabetes and upregulated in those with obesity or impaired fasting glucose. However, gender-balanced data in older populations, in whom risk factors commonly culminate in overt cardiovascular disease, are scarce. We assessed in non-diabetic Caucasian subjects with stage I-II obesity in the early aging range (50 to 70 years), the relationship between irisin, body composition and markers of metabolic derangement by gender. In 60 (31 women, 29 men) non-diabetics with a body mass index ≥30 - ≤40 kg/m^2^, we measured anthropometrics and body composition (Air Displacement Plethysmography). We assayed lipid and glucose profile by routine methods, plasma irisin by ELISA and measured insulin resistance by the HOMA index. Irisin levels were higher in women than in men (161 [105–198]) vs 83 [33–115] ng/ml, P<0.001), and correlated directly with HOMA index in both (rho 0.735, P<0.001 M, rho 0.452, P = 0.011 F). Sex differences were maintained across insulin resistance severity stages. In men, irisin concentrations correlated directly with body mass index (rho 0.755, P<0.001), waist circumference (rho 0.623, P<0.001), fat mass index (rho 0.762, P<0.001), glucose (rho 0.408, P = 0.028), the fatty liver index (rho 0.705, P<0.001) and FINDRISC score (rho 0.536, P = 0.003). Among non-diabetic Caucasian subjects with obesity in the early stages of aging, irisin levels reflect the amount of body fat and insulin resistance severity, independently of between-gender differences in the adipomyokine concentrations and are associated with markers of visceral adiposity in men but not in women.

## Introduction

Irisin is a recently discovered [1] myokine secreted in the skeletal muscle in response to physical activity, that has been shown to promote browning of the white adipose tissue [WAT), thermogenesis, energy expenditure and consumption of lipid reserves [2].

The WAT is the second main source of circulating irisin concentrations [3]. In obese subjects (OBs) irisin levels seem to directly reflect the amount of WAT, independently of physical activity and energy expenditure [4]. Weight loss by energy restriction has been shown to decrease circulating levels of irisin, with an eventual increase if weight is regained [5]. Irisin concentrations were found to be positively associated with adiposity indexes including body mass index (BMI), waist circumference, waist-to-hip ratio, and fat mass in children [6], premenopausal women [4], middle-aged and young elderly men [7], but not in young-elderly women [8].

While concentrations of the adipomyokine were found to be decreased in subjects with overt diabetes [9], in non-diabetics the relationship between irisin levels and metabolic abnormalities has long been controversial. A recent meta-analysis [10] evaluated 17 studies that assessed irisin in relation to insulin resistance in populations of different geographic origin; all studies included subjects younger on average than 60 years and half were limited to a single gender assessment. The meta-analysis concluded that in non-diabetics circulating irisin was significantly and positively associated with insulin resistance (IR) as measured by the Homeostasis Model Assessment (HOMA) index, with a possible influence of sex and ethnicity on the correlation coefficient. Although the association was reported to be independent of assay method, BMI and age, the cohorts included in the meta-analysis were mostly young or middle-aged. More recently, upregulation in circulating irisin among OBs was found to be exacerbated in individuals with impaired fasting glucose or diabetes [7]. Few data are available in older populations, at an age when cardiovascular (CV) risk factors more commonly culminate in overt CV disease.

Aims of our study are to assess the correlation between irisin and markers of metabolic derangement during the early stages of aging (age range 50–70 years) in Caucasian OBs and to evaluate the impact of gender on this relationship.

## Materials and methods

The protocol was approved by Niguarda Hospital Institutional Ethics Committee. The study was performed in accordance with the ethical standards laid down in the 1964 Declaration of Helsinki and its later amendments. All participants signed an informed consent prior to enrolment.

### Study population

Subjects aged >50 and ≤70 years with stage I and II OB (BMI≥30 and <40 kg/m^2^) followed-up at the Nutrition Division of our Institution between June 2017 and June 2018 were screened for participation. We excluded patients with stage IV chronic kidney dysfunction (estimated glomerular filtration rate <15 ml/min), liver dysfunction (clinically diagnosed liver disease or new finding of elevated AST-ALT x 2 the upper normalcy range from our laboratory), active cancer (diagnosed <5 years before enrolment and/or imaging or laboratory evidence of recurrence), claustrophobia and psychiatric morbidity or any other condition that might impair the ability to give informed consent. Subjects with overt diabetes (fasting glucose ≥126 mg/dl or glycated hemoglobin (HbA1c) ≥48 mmol/mol) were also excluded, whereas those with impaired fasting glucose (100–125 mg/dl) or borderline HbA1c values (43–47 mmol/mol) were included.

Subjects were on a group weight management program tailored to consolidation and maintenance of weight loss through a dietary regimen combined with lifestyle advice. The diet was based on the principles of the Modern Mediterranean Diet Pyramid modified to avoid high caloric food and distributed in 3 main meals and 2 healthy snacks during the day. Caloric restriction was tailored to baseline resting energy expenditure measured by indirect calorimetry. To avoid catabolic processes, caloric intake was adjusted to the physical activity level by a 1.2–1.3 factor.

We classified subjects based on their PAL as sedentary (<1.70), moderately active (1.70–1.99), vigorously active (≥2), according to "Human energy requirements: Energy Requirement of Adults". Report of a Joint FAO/WHO/UNU Expert Consultation 2004.

Lifestyle advice encouraged light to moderate physical activity consisting of 30 minutes of moderate aerobic exercise (brisk walking) 4 times/week.

During the monthly group meetings led by a physician and the dietitian, adherence to the prescribed regimen was checked by anthropometric measurements and personal interviews.

None of the enrolled subjects had lost more than 5% of their body weight during the two months prior to enrolment.

### Study design

Eligible consenting subjects attended our institute in the fasting state and underwent medical history recording, anthropometric measures (height, weight, BMI as weight kg/height m^2^, waist circumference measured at the midpoint between the lower border of the rib cage and iliac crest), body composition, measurement of tissue Advanced Glycation End-products (AGE), blood and urine sampling, the Finnish Diabetes Risk Score (FINDRISC) questionnaire and the Fatty Liver Index (FLI).

### Blood samples

Peripheral blood samples (10 ml) were collected in the fasting state using one serum separator and two EDTA vacutainer tubes. One blood in EDTA was immediately centrifuged at 4000 rpm, 4°C for 10 minutes in order to obtain plasma aliquots. Plasma samples were than stored at -80°C before irisin assessment.

Blood, collected in serum separator tube, was kept at room temperature for 30 minutes to allow sample coagulation before centrifugation at 4000 rpm for 15 minutes. Serum and the other EDTA sample were immediately used for routine biochemistry tests (glucose, HbA1c, insulin, total/HDL/LDL cholesterol, triglycerides) which were assayed by standard laboratory procedures.

HOMA index, was calculated as follows: (fasting glucose mg/dl x fasting insulin μU/ml)/405 and classified in 3 categories <2.5 (no IR), 2.5–3.8 (mild-moderate IR), >3.8 (definite IR), based on a cut-off validated in Mediterranean populations [11].

### Measurement of plasma irisin concentrations

Plasma irisin levels were determined in duplicate, using a commercial ELISA kit (CSB-EQ027943HU, Cusabio Life Science, Inc.) which employs the quantitative sandwich enzyme immunoassay technique. Assay sensitivity was 0.78 ng/ml and detection range was 3.12–200 ng/ml. The intra- and inter- assay variations were both less than 10%.

Reference irisin levels for the OBs age group studied were obtained by simultaneous assay of age- and gender-matched control plasma samples, stored at our laboratory, from healthy non obese (BMI 25 [24–27 kg/m^2^]) subjects.

### Functional testing

AGEs were measured on the volar side of the forearm, 10 cm below the elbow, at room temperature using the AGE Reader® (DiagnOptics Technologies BV, Groningen, the Netherlands) device. Skin autofluorescence was calculated as mean of two measurements and results were presented as an absolute numerical value. Reference normative values were derived from [12].

### Measurement of the body composition

Body composition was assessed by Air Displacement Plethysmography (BOD POD®, Cosmed), from an average of six volume measurements. From the subject’s body density, relative proportions of body fat and lean body mass were calculated using Siri equation: % Fat = [495/Density] ‐ 450 and % Lean Body Mass = 100 ‐ % Fat [13]. Fat free mass index was estimated as (body weight kg x fat-free mass/100)/height m^2^.

### The FINDRISC questionnaire

The FINDRISC tool is a 8-item questionnaire, that estimates the probability a person has to develop type 2 diabetes within the next 10 years, based on age, family history, anthropometrics (BMI, WC), vegetable intake, physical activity, history of hypertension and/or hyperglycemia; it has a maximum value of 26 [14].

### The FLI

The FLI, an algorithm based on BMI, waist circumference, triglycerides and gamma-glutamyl transferase is one of the best-validated markers for liver steatosis [15]. FLI has been documented to be associated with visceral adiposity and cardiometabolic risk.[16–17].

### Metabolic syndrome

The metabolic profile was determined from a multicomponent cardiometabolic risk score: waist circumference ≥88 cm in women and ≥100 cm in men, systolic/diastolic blood pressure ≥130/85 mmHg, HDL cholesterol <50 mg/dl in women and <40 mg/dl in men, triglycerides ≥150 mg/dl, fasting plasma glucose ≥100 mg/dl. According to the NCEP-III criteria [18], subjects were considered to have the metabolic syndrome (MetS) if at least 3 of the 5 above parameters were present.

### Statistical analysis

Enrolment was based on the estimated proportion of subjects presenting a HOMA-IR>3.8. A group sample size of 60 patients achieved >80% power with alpha = 0.05 to detect a 30% (95% CI 0.18–0.42) proportion of HOMA-IR>3.8 in the study population.

Data are presented as median and interquartile range [I-III] for continuous variables or number and percentage for categorical variables. Normality assumptions of variable distributions were examined by Shapiro-Wilk's test. Between-group differences were tested by Student’s t-test for continuous variables, or by Mann-Whitney test for skewed data, and by chi-square test or Fisher exact test for categorical variables. The relationships between irisin and other clinical and biochemical parameters were tested using Spearman’s rank correlation coefficient. Statistical analyses were performed using SPSS ver. 24.0 software package (IBM SPSS, New York, USA). A P-value <0.05 was considered significant.

## Results

Among 218 screened subjects, 60 (31 females, 29 males) had no exclusion criterion and consented to participate in the study. Table 1 depicts the anthropometric and metabolic characteristics of the enrolled population categorized by gender.

**Table 1:** Comparison by gender.

	Women N = 31	Men N = 29	P
Age, years	59 [57–64]	59 [55–63]	0.389
Cardiovascular risk factors			
Smoking habit, n (%)	2 (7%)	7 (24%)	0.076
Hypertension, n (%)	13 (42%)	19 (66%)	0.077
Dyslipidemia, n (%)	12 (39%)	10 (35%)	0.793
Anthropometric mesures			
BMI, kg/m^2^	33 [32–35]	35 [32–37]	0.149
Waist circumference, cm	103 [97–106]	120 [109–124]	**<0.001**
Body composition			
Fat mass, %	51 [47–54]	41 [35–44]	**<0.001**
Fat free mass, %	49 [46–53]	59 [56–65]	**<0.001**
Fat/free fat ratio	1.03 [0.88–1.15]	0.71 [0.54–0.80]	**<0.001**
Fat mass index, kg/m^2^	16.3 [15.3–18.9]	14.6 [10.9–16.7]	**<0.001**
Fat-free mass index, kg/m^2^	16.6 [15.8–17.6]	21.0 [19.2–21.8]	**<0.001**
Vital signs			
Systolic blood pressure, mmHg	110 [105–120]	120 [113–130]	**0.010**
Diastolic blood pressure, mmHg	70 [60–70]	70 [70–80]	**0.008**
Heart rate, beat/min	60 [56–68]	62 [55–69]	0.930
Creatinine, mg/dl	0.74 [0.65–0.79]	1.00 [0.85–1.07]	**<0.001**
Lipid profile			
Total cholesterol, mg/dl	214 [188–241]	190 [180–216]	**0.028**
HDL cholesterol, mg/dl	56 [51–65]	44 [38–51]	**<0.001**
LDL cholesterol, mg/dl	133 [113–150]	120 [108–144]	0.204
Triglycerides, mg/dl	100 [81–121]	110 [84–145]	0.391
Glucose profile			
Glucose, mg/dl	95 [90–110]	98 [95–107]	0.662
Glycated Hemoglobin, mmol/mol	37 [35–41]	36 [35–39]	0.334
Insulin, μU/ml	11.8 [7.8–18.8]	13.8 [10.1–21.0]	0.325
HOMA Index	2.53 [1.80–5.11]	3.41 [2.37–5.53]	0.348
HOMA index class			0.366
<2.5	14 (45%)	8 (28%)	
2.5–3.8	6 (19%)	7 (24%)	
>3.8	11 (36%)	14 (48%)	
AGE score	2.23 [2.10–2.50]	2.25 [2.05–2.60]	0.918
FINDRISC	14 [12–17]	14 [12–17]	0.817
FLI	64 [55–77]	92 [79–94]	**<0.001**
Irisin, ng/ml	161 [105–198]	83 [33–115]	**<0.001**

Data are expressed as number (%) and median and interquartiles [I-III].

AGE, Advanced Glycation End-products; BMI, Body Mass Index; FLI, Fatty Liver Index; FINDRISC, Finnish Diabetes Risk Score; HDL, High-Density Lipoprotein; LDL, Low-Density Lipoprotein; HOMA, Homeostasis Model Assessment.

Waist circumference, fat-free mass (both expressed as % or kg/m^2^), blood pressure, creatinine concentrations, and FLI were significantly higher in men than in women. Conversely, fat mass (% or kg/m^2^), fat/fat-free mass ratio, total and HDL cholesterol concentrations, were significantly higher in women than in men.

Irisin levels were higher in our OBs compared to those obtained in plasma samples from healthy non OB controls (107 [80–179] versus 52 [20–74] ng/ml, P<0.001, respectively). Moreover, irisin values were higher in women than in men both in controls (71 [50–90] vs 21 [5–53] ng/ml, P = 0.009) and in study patients (161 [105–198] vs 83 [33–115] ng/ml, P<0.001).

**Fig 1:**
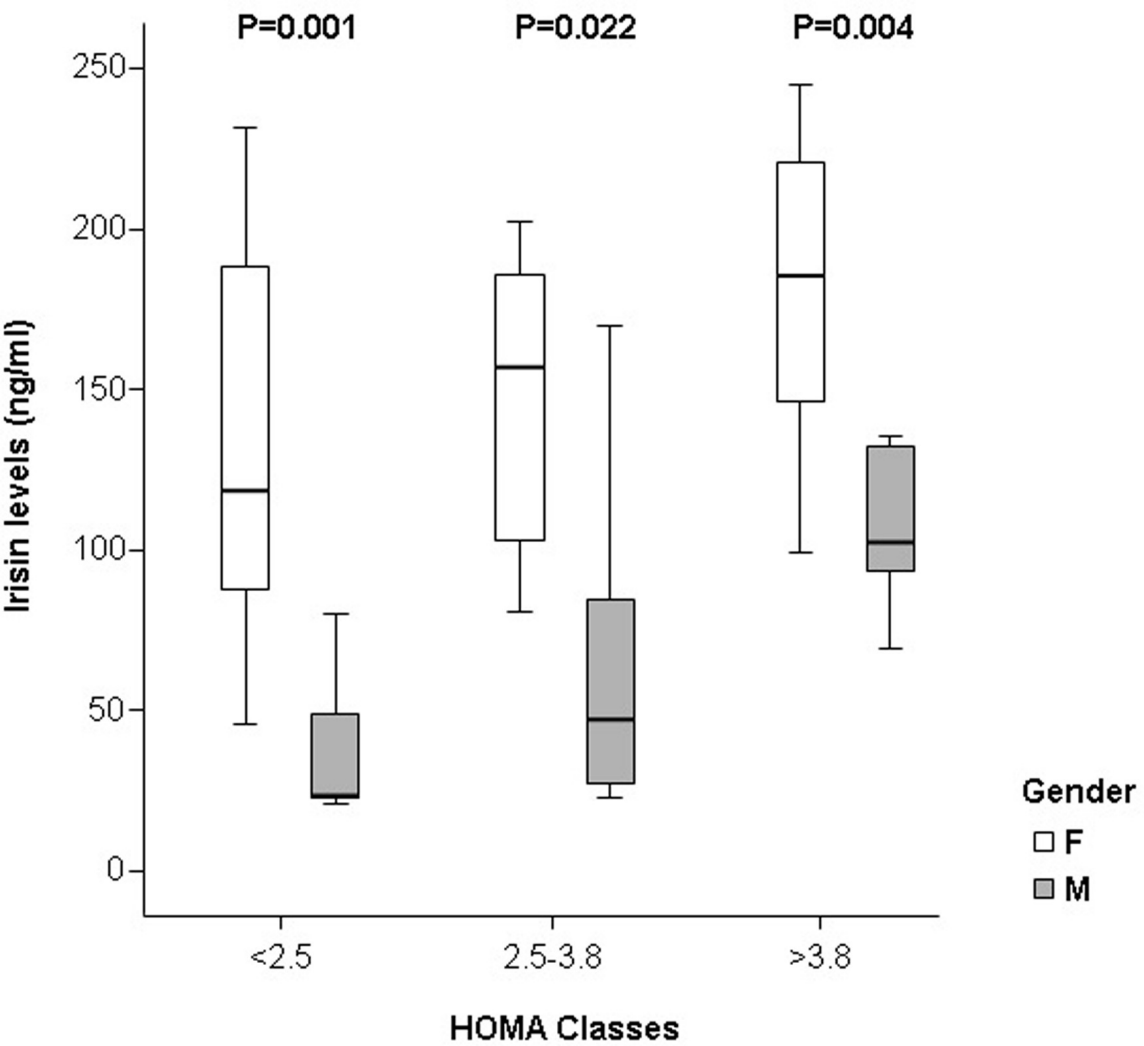
Box-plots of irisin concentrations by gender and homeostasis model assessment (HOMA) index categories: Irisin increases significantly with severity of insulin resistance in both women and men.

In both genders, irisin concentrations correlated directly (Table 2) with insulin and HOMA index. Irisin levels increased with the severity of IR (in the overall population P = 0.041) as expressed by HOMA index categories, in both genders, but were always significantly higher in women than in men (Fig 1).

**Table 2:** Correlations of circulating serum irisin with different parameters by gender.

	Women N = 31	P value	Men N = 29	P value
Age, Years	-0.257	0.162	0.163	0.398
BMI, kg/m^2^	0.129	0.488	0.755	**<0.001**
Waist circumference, cm	-0.225	0.224	0.623	**<0.001**
Fat mass, %	0.215	0.245	0.704	**<0.001**
Fat free mass, %	-0.234	0.205	-0.704	**<0.001**
Fat/fat free mass ratio	0.215	0.245	0.704	**<0.001**
Fat mass index, kg/m^2^	0.239	0,196	0.762	**<0.001**
Fat-free mass index, kg/m^2^	-0.100	0.591	0.216	0.261
Systolic blood pressure, mmHg	-0.219	0.236	0.043	0.826
Diastolic blood pressure, mmHg	-0.116	0.534	-0.052	0.790
Heart rate, beat/min	0.035	0.850	0.326	0.084
Creatinine, mg/dl	-0.038	0.847	0.151	0.433
Total cholesterol, mg/dl	-0.077	0.680	-0.175	0.365
HDL cholesterol, mg/dl	0.129	0.490	-0.099	0.608
LDL cholesterol, mg/dl	-0.099	0.598	-0.105	0.587
Triglycerides, mg/dl	-0.273	0.137	0.017	0.932
Glucose, mg/dl	0.286	0.118	0.408	**0.028**
Glycated Hemoglobin, mmol/mol	0.053	0.779	0.275	0.148
Insulin, μU/ml	0.424	**0.017**	0.743	**<0.001**
HOMA Index	0.452	**0.011**	0.735	**<0.001**
AGE	-0.052	0.787	0.182	0.344
FINDRISC	0.143	0.441	0.536	**0.003**
FLI	-0.001	0.995	0.705	**<0.001**

AGE, advanced glycation end-products; BMI, body mass index; FINDRISC, Finnish diabetes risk score; FLI, fatty liver index; HDL, high density lipoprotein; HOMA, homeostasis model assessment; LDL, low density lipoprotein.

Irisin concentrations correlated directly (Table 2) with BMI, waist, fat mass, both percent and indexed, glucose, FINDRISC score and FLI in men only. No correlations were found between irisin and age, lipid concentrations, blood pressure values and skin autofluorescence.

Irisin concentrations did not differ according to the presence or absence of the MetS either in men (MetS 94 [75–126]; no MetS 51 [23–107] ng/ml, P = 0.076) or in women (MetS 149 [124–207]; no MetS 174 [95–199] ng/ml, P = 0.667).

**Fig 2:**
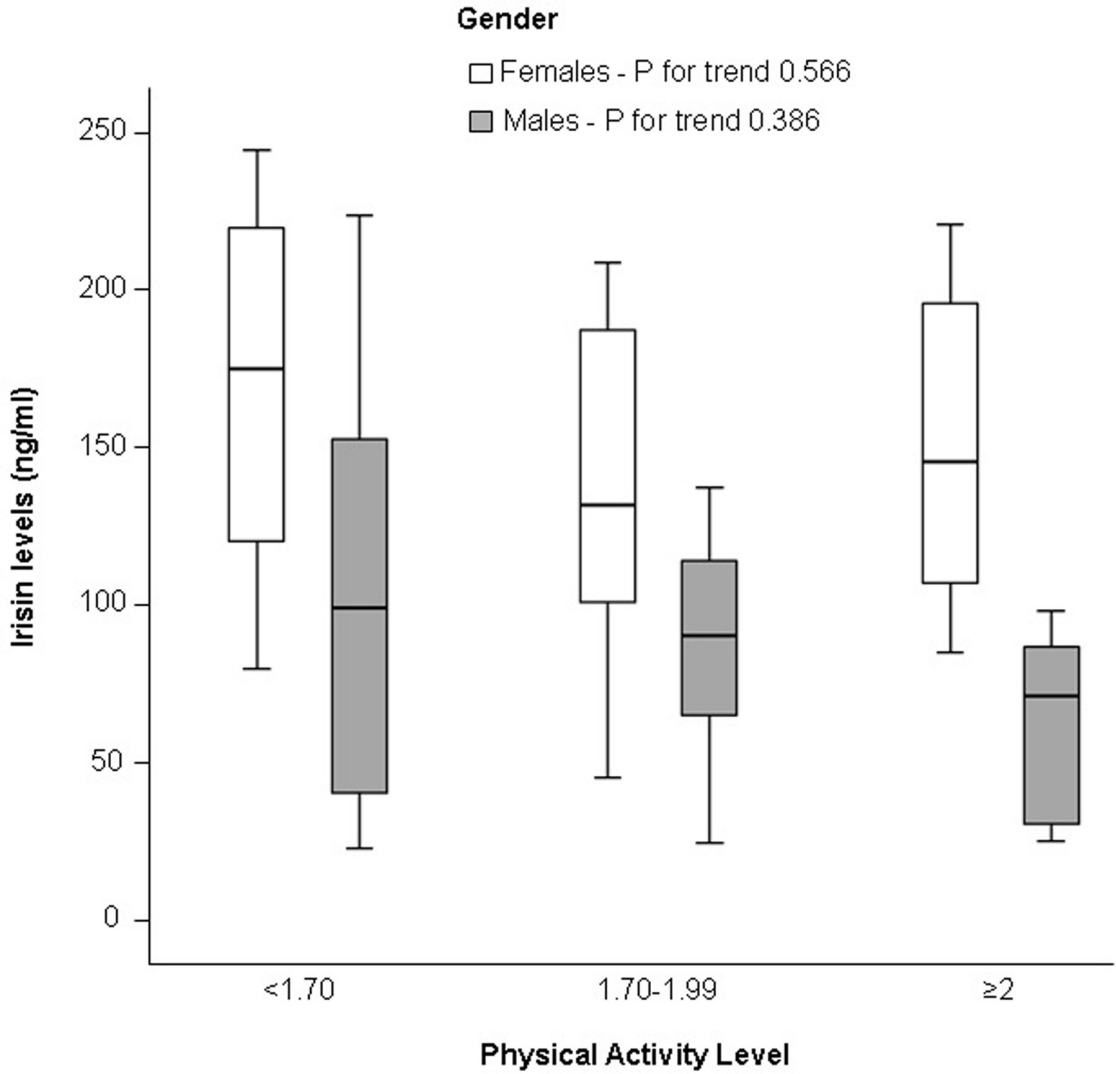
Box-plots of irisin concentrations by gender and physical activity level (PAL) categories. We classified subjects based on their PAL as sedentary (<1.70), moderately active (1.70–1.99) and vigorously active (≥2).

Based on their PAL, 27 subjects (12 males and 15 females) were sedentary, 19 were moderately active (8 males and 11 females) and 14 were vigorously active (9 males and 5 females). PAL was not associated with irisin concentrations either in men or in women (Fig 2).

## Discussion

Among non-diabetic OB Caucasians in the early aging stage, irisin levels are higher in women than in men, directly correlate with IR measures in both genders, but show gender-divergent associations with measures of body composition and metabolic derangement.

Although previous studies on circulating irisin have been conflicting, the balance of evidence supports the notion that in OBs irisin levels are increased in parallel to expanded body fat [2].

Gender-differences in irisin levels have been observed: women consistently displaying higher values than men among Saudi children [19], middle-aged Chinese [20] and Japanese adults [21]. The divergent results from studies performed in healthy, non OB, and young population [22, 23] may be ascribed to the physiologically higher muscle mass in males compared to females, in the absence of increased excess body fat. Comparative data across genders in elderly Caucasian have not been reported. Gender disparity has been suggested to depend on fat distribution in women and the anabolic effects of estradiol [4]. The post-menopausal condition may further contribute to plasma increase of irisin levels, as reported by Zügel et al [24]. Our OB post-menopausal women showed almost two-fold irisin concentrations as compared to men of comparable age; the trend was similar in assays from our reference age-matched population.

We did not observe a relationship between irisin and adiposity indexes and markers of visceral fat (waist circumference, FLI) in our post-menopausal women. Conversely, indicators of visceral adiposity, that have been consistently associated to increased CV risk beyond glucose profile derangements, correlated with the adipomyokine levels in men only. The distribution of fat to the legs and trunk has recently shown differential genetic associations between sexes: a greater capacity for peripheral adipose storage in women parallels a lower risk of CV and metabolic disease [25]. However, irisin levels did not differ with respect to the presence of MetS in both gender groups, as previously reported in younger subjects with a wider range of glucose tolerance and BMI levels [26].

Conversely, in our Caucasian cohort in the early stages of aging, the association between irisin, insulin levels and HOMA index was significant in both genders, in agreement with previous reports in younger Asian populations [21] and with the meta-analysis by Qiu et al [10]. In our series, sex differences were maintained across IR severity stages. Our findings support the hypothesis that irisin levels in OBs may increase as compensatory mechanism to augment energy expenditure, to improve insulin sensitivity and maintain metabolic homeostasis [27].

The tissue accumulation of AGEs, as measured by skin autofluorescence, correlates with carotid intima-media thickness and endothelial dysfunction and has been associated with diabetic complications, cardiovascular mortality and the degree of atherosclerosis [28]. Li et al. [29] observed an inverse correlation between AGEs and irisin levels in type 2 diabetics, but no data have been reported for non-diabetic OBs. In a large cohort study, van Waateringe et al.[30] showed that BMI was weakly associated with skin autofluorescence values after adjustment for age, renal function, cigarette packs smoked and coffee consumption. Our findings show that, in a homogeneous population of non-diabetic subjects with mild-to-moderate obesity, irisin levels are not related to AGEs, despite their association with IR.

The relationship between irisin level and chronic exercise training or habitual physical activity is controversial [31]: both increasing [1,32] and decreasing [4,33] circulating irisin concentrations have been observed. In agreement with the results of the KarMeN study, that correlated irisin concentrations with both subjective and objective measures of physical activity in healthy, normal weight subjects [34], we found no association between irisin and habitual physical activity in our patients examined under resting condition.

The discrepancies between the results of the existing studies on circulating irisin levels in human may be partly attributed to the characteristic of the enrolled populations (ethnicity, age, gender etc), but also to methodological differences. Five different irisin assays have been described in the literature [35]: the level of irisin in serum and plasma reported by using these analytical assays ranged from 1 to 2000 ng/ml. The presence of different protein fragments, protein glycosylation, the presence of free or complexed protein forms in the biological samples and the cross reactivity of antibodies with other proteins, may all alter the results of these immunoassays. We used the same Cusabio ELISA kit batch, to measure plasma irisin both in OB subjects and in non OB controls.

Our study has several limitations. The study cohort, though homogeneous for ethnicity and exclusion of diabetics, was relatively small, hence our findings need to be confirmed in a larger population. However, we conducted our study in a gender-balanced sample, in an age range rarely addressed in the literature on the topic. We restricted enrolment to stage I and II OB, and the lack of extreme BMI values may have limited the ability to detect variations in irisin with fat-free mass. However, consistent variations in IR were found that were mirrored by changes in irisin concentrations.

In conclusion, among our non-diabetic Caucasian subjects with stage I and II OB, in the early aging range, irisin levels reflect the amount of body fat and the severity of IR, independently of between-gender differences in the adipomyokine concentrations. Irisin concentrations correlates with markers of visceral adiposity in men but not in women.

## Supporting information

S1 File (DOC)
