# Supplementary material for: Irisin and markers of metabolic derangement in non-diabetic Caucasian subjects with stage I-II obesity during early aging
Source: PLoS One. 2020 Feb 18;15(2):e0229152. doi: 10.1371/journal.pone.0229152 (PMC7028288; doi:10.1371/journal.pone.0229152)
Supplement: S1 File — (DOC) [file pone.0229152.s001.doc]

**Supporting information file**

**Fig 1. I**risin concentrations percentiles by gender and Homeostasis Model Assessment (HOMA) index categories.

Women

| **HOMA Index Classes** | **Irisin ng/ml (25th)** | **Irisin ng/ml (50th)** | **Irisin ng/ml (75th)** |
| --- | --- | --- | --- |
| < 2.5 (N=14) | 87 | 118 | 190 |
| 2.5-3.8 (N=6) | 97 | 157 | 190 |
| > 3.8 (N=11) | 142 | 185 | 223 |

Men

| **HOMA Index Classes** | **Irisin ng/ml (25th)** | **Irisin ng/ml (50th)** | **Irisin ng/ml (75th)** |
| --- | --- | --- | --- |
| < 2.5 (N=8) | 22 | 23 | 51 |
| 2.5-3.8 (N=7) | 25 | 47 | 85 |
| > 3.8 (N=14) | 90 | 102 | 133 |

**Fig 2.** **I**risin concentrations percentiles by gender and PAL classes**.**

Women

| **PAL Classes** | **Irisin ng/ml (25th)** | **Irisin ng/ml (50th)** | **Irisin ng/ml (75th)** |
| --- | --- | --- | --- |
| < 1.70 (N=15) | 119 | 175 | 223 |
| 1.70-1.99 (N=11) | 99 | 132 | 194 |
| > 1.99 (N=5) | 96 | 146 | 209 |

Men

| **PAL Classes** | **Irisin ng/ml (25th)** | **Irisin ng/ml (50th)** | **Irisin ng/ml (75th)** |
| --- | --- | --- | --- |
| < 1.70 (N=12) | 25 | 97 | 169 |
| 1.70-1.99 (N=8) | 55 | 89 | 115 |
| > 1.99 (N=9) | 26 | 69 | 89 |
